# The 9th Barossa Meeting: Cell Signalling in Cancer Medicine

**DOI:** 10.1038/s41419-020-2364-9

**Published:** 2020-03-02

**Authors:** Loretta Dorstyn, Denis Tvorogov, Shiva Malek, Nirmal Robinson

**Affiliations:** 1 Centre for Cancer Biology, University of South Australia and SA Pathology, Adelaide, SA 5000 Australia; 2 Department of Discovery Oncology, Genentech, Inc., 1 DNA Way South San Francisco, San Francisco, CA 94080 USA

**Keywords:** Cancer, Cancer

Organized by the Center for Cancer Biology (Adelaide, Australia), the 9th Barossa Meeting on “Cell Signalling in Cancer Medicine” was held in November 2019 in one of the most historic wine-producing regions in Australia, the Barossa Valley. This biennial meeting brought together eminent international and national leaders, ECRs, and students and provided a unique opportunity for interaction in a relaxing environment surrounded by vineyards, premium cellar doors, and exceptional food (Fig. 1a). The focus of discussion was future directions in cancer biology combined with the challenges and developments in cancer medicine.

**Fig. 1:**
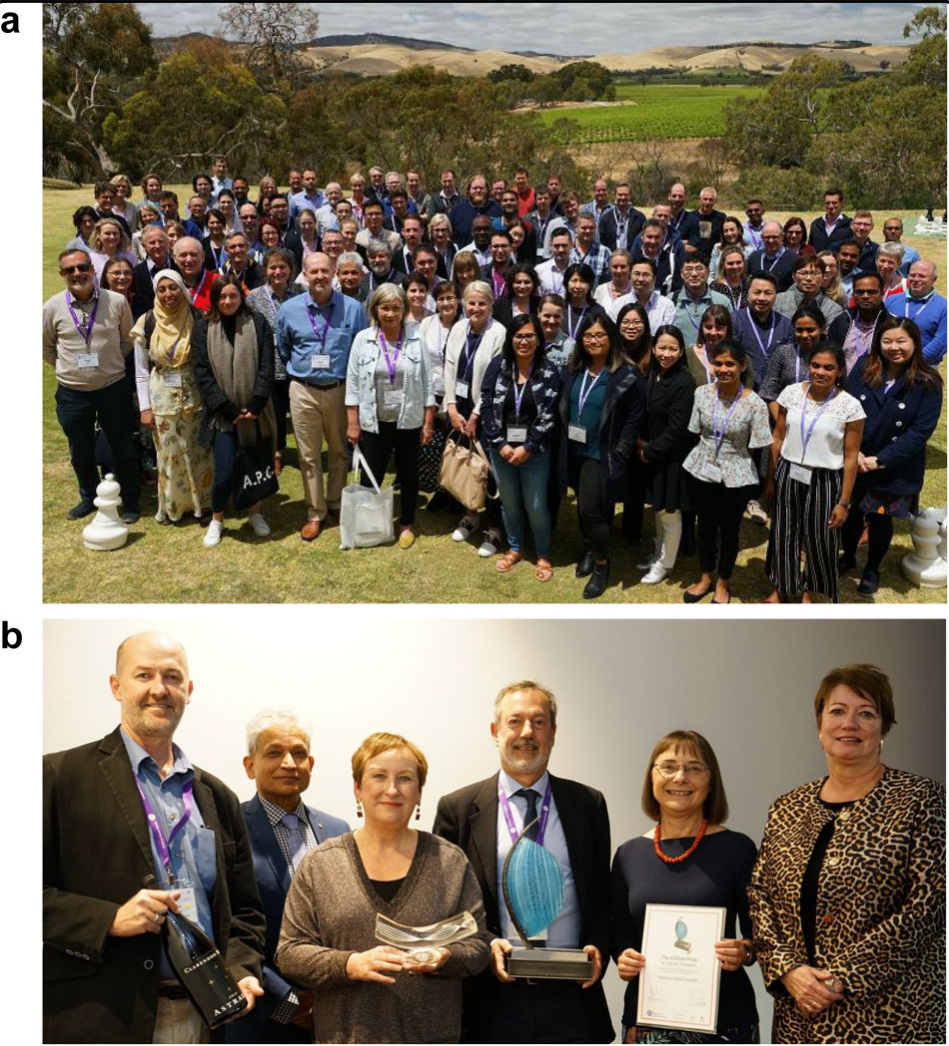
The 9th Barossa Meeting. **a** Delegates of the meeting. **b** 2019 Clifford Prize for Cancer Research awarded to Prof. Karen Vousden, The Francis Crick Institute, UK (second from right) by (left to right) Professor Stuart Pitson (Centre for Cancer Biology, Adelaide, Australia), Professor Sharad Kumar (Centre for Cancer Biology, Adelaide, Australia), Ms Lesley Dwyer (CEO, Central Adelaide Local Health Network), Professor Angel Lopez (Centre for Cancer Biology, Adelaide, Australia) and Ms Julie Hartley-Jones (Central Adelaide Local Health Network).

## Tumor microenvironment

The importance of the tumor microenvironment in cancer progression and metastasis was the theme that kicked off the meeting. An elegant talk by Erik Sahai (The Francis Crick Institute, UK), who discussed how the spread of breast cancer cells into other tissues is often followed by extended periods of cancer cell dormancy and latency of metastasis. He discussed how lung epithelium influences metastatic growth of indolent breast cancer cells and identified several latency genes that drive cell survival. Such mechanisms underlying cancer cell dormancy and metastatic growth are important to enable alternative treatment strategies.

Tumor stroma remodelling in cancer progression and metastasis was highlighted by Thomas Cox (Garvan Institute of Medical Research, Australia), who discussed efforts to reduce desmoplasia in cancer, a particular feature of pancreatic ductal adenocarcinoma associated with chemoresistance. This has led to the development of a small-molecule inhibitor of lysyl oxidases, key players of extracellular matrix (ECM) biogenesis, which are overexpressed in many solid tumors and associated with poor survival. This was followed by Michael Samuel (Center for Cancer Biology, Australia), who discussed the role of activated ROCK (Rho-associated protein kinase) in ECM production and demonstrated ROCK-mediated ER (endoplasmic reticulum) stress response in educating fibroblasts as a key determinant of ECM signalling and tumor progression.

## Cancer signalling and mechanisms

A focus of the cancer signalling theme was the importance of ubiquitination, autophagy, and oxidative stress that drive cellular homeostasis and cancer progression. One of the highlights was a talk by Ivan Dikic (Goethe University, Germany), who described the identification of alternative mechanisms that regulate the ubiquitin system and autophagy, by studying the pathogenesis of *Legionella pneumophila*. In particular, the SidE family of ubiquitin ligases in *Legionella* that mediate atypical serine ubiquitination, implicated to have a role in ER-phagy and mitochondrial metabolism. The importance of the ubiquitin system in cancer was further highlighted by David Komander (Walter and Eliza Hall Institute of Medical Research, Australia), who described linkage specificity and targeting of deubiquitinases (DUBs) that determine physiological signalling outcome. The identification of several DUB inhibitors, including inhibitors of USP28, which regulate various oncogenes (e.g., c-Myc) and USP25, which regulates TRAF (tumor necrosis factor receptor-associated factor) signalling, provide alternative approaches to induce degradation of potential “un-druggable” molecules in cancer.

Gerry Melino (University of Cambridge, UK) presented data demonstrating the p53 family protein TAp73, facilitates ubiquitin-dependent degradation of HIF1α, thereby suppressing tumor progression. Seminal work on the molecular mechanisms of p53 in cancer was elaborated by Karen Vousden (The Francis Crick Institute, UK), the worthy recipient of the 2019 Clifford Prize for Cancer Research (Fig. 1b). She also discussed the importance of metabolic pathway regulation and balanced levels of reactive oxygen species (ROS) in the control of cancer cell survival. In particular, the antioxidant role of TP53-induced glycolysis regulatory phosphatase (TIGAR) in this process, which is overexpressed in cancers and is associated with poor prognosis. Their findings demonstrated that both the levels and timing of ROS are critical factors to consider for the better treatment of tumors.

## Cutting-edge approaches and targets in cancer therapy

In a national initiative that aims to provide personalized treatment for pediatric cancers, Paul Eckert (Children’s Cancer Institute, Australia) spoke of the “National Zero Childhood Cancer (ZERO) Program.” Genomic analysis has provided much understanding of mechanisms that drive high-risk childhood cancers, including identification of complex genetic and novel oncogenic events that will help model response to therapy and provide better targeted therapy. As an additional approach to monitor disease progression, Sarah-Jane Dawson (Peter MacCallum Cancer Centre, Australia) discussed how analyzing circulating DNA has identified specific gene mutations, expression profiles, and metastatic markers associated with treatment response and resistance to enable targeted treatment combinations to reduce metastatic disease. Belinda Parker (Peter MacCallum Cancer Centre, Australia) also spoke of identifying biomarkers that predict metastasis for precision immunotherapies in prostate cancer. The identification of interferon-1 (IFN-I)-mediated intratumoral immune changes has suggested approaches that stimulate IFN signalling can prevent metastatic bone disease.

Charles de Bock (Children’s Cancer Institute, Australia) discussed the challenges of treating T cell acute lymphoblastic leukemia (T-ALL), associated with multiple mutations, and presented new data demonstrating improved efficacy through selective Psen1 inhibition. Identification of novel gene fusions as drivers of immature acute T-ALL, associated with activating NRAS mutations, provide alternative targets for personalized therapies. Shiva Malek (Genentech Inc., USA) presented clinical data using a pan-Raf kinase inhibitor that has shown promise in treating NRAS and BRAF^V600E^ mutant tumors. Importantly, mechanisms of resistance to pan-RAF inhibition have been identified as biomarkers to improve treatment efficacy.

There were several stimulating talks describing the identification of potential new drivers and targets for therapy. Madelon Maurice (Oncode Institute, Netherlands) described the mechanisms of loss-of-function mutations in ZNRF3/RNF43 that drive Wnt hypersensitivity and tumor growth. This has led to the development of single-chain antibody against LRP6 that can selectively block Wnt binding and growth of intestinal organoids. Her work also described the importance of mutational screening for Wnt-based therapies. Jo Woodcock (Centre for Cancer Biology, Australia) discussed the role of the 14-3-3 family of proteins in oncogenic signalling with a focus on non-small-cell lung cancer (NSCLC) and described the generation of drugs that disrupt 14-3-3 dimer dynamics that effectively reduce growth of NSCLC xenografts.

## Tumor heterogeneity

Tuomas Tammela (Memorial Sloan Kettering Cancer Centre, USA) described targeting plasticity in cancer as a promising treatment strategy to overcome resistance. Tammela’s group identified distinct lung cell populations associated with cancer cell evolution, including a high plasticity cell state that harbored aggressive features and correlated with resistance and worse survival. This suggests that the ability to alter cell state has therapeutic potential. David Croucher (Garvin Institute of Medical Research, Australia) described a distinct chemoresistant single-cell population in high-risk neuroblastoma, associated with reduced c-Jun N-terminal kinase signalling and apoptosis defect. Importantly, this work demonstrated potential for targeting these cells by inhibiting both MCL1 and Bcl-2. The degree of tumor heterogeneity in neuroblastoma was further highlighted by Bengt Hallberg (University of Gothenburg, Sweden) with the identification of a novel ALK mutation and ALKAL2 overexpression that cooperate with MYCN to drive aggressive disease has provided promise for treatment with ALK inhibitors.

## Hematological cancers

A theme dedicated to hematopoietic malignancies began with an inspiring talk by Lucy Godley (University of Chicago, USA), who discussed how next-generation sequencing approaches and molecular disease profiling have identified germline mutations, new single-nucleotide variants, gene duplications, and deletion events that contribute to disease progression, particularly in myelodysplastic syndrome (MDS). Importantly, the age of disease presentation is a surrogate for changes in biological pathways (DNA damage, DNA repair) and new germline mutations in DDX41 that effect its role in cGAS/STING signalling are associated with increased risk of MDS and acute myeloid leukemia (AML).

The genetic and molecular mechanisms associated with MDS was further discussed by Brian Bath (University of New Hampshire, USA), who showed that targeting GDF1 could increase intracellular ceramide levels and restore effective hematopoiesis, as an alternative treatment strategy in MDS and AML. Following on from this theme, Jason Powell (Centre for Cancer Biology, Australia) discussed novel efforts to target sphingosine kinase-1 to induce a ceramide-dependent apoptosis integrated stress response in AML. Daniel Thomas (SAHMRI and University of Adelaide, Australia) then discussed the challenges of precision oncology, given that many newly found mutations are not druggable. He described new bioinformatics methods for drug repurposing based on differentiation profiles and the identification of mutation-specific synthetic lethal therapies from copy number changes, including novel metabolic targets for IDH1 mutations in AML.

The meeting presented cutting-edge research in cancer biology and strategies to treat specific cancers, while discussing the existing challenges toward precision medicine. Advances in omics profiling, genomics, and imaging techniques combined with in vivo models of disease have made possible the development of new drug targets as well as repurposing drugs for better treatment options. It is anticipated the next Barossa meeting, in November 2021, will provide much exiting new insights into cancer medicine.

